# Supplementary figures and images for: Evolinc: A Tool for the Identification and Evolutionary Comparison of Long Intergenic Non-coding RNAs
Source: Front Genet. 2017 May 9;8:52. doi: 10.3389/fgene.2017.00052 (PMC5422434; doi:10.3389/fgene.2017.00052)

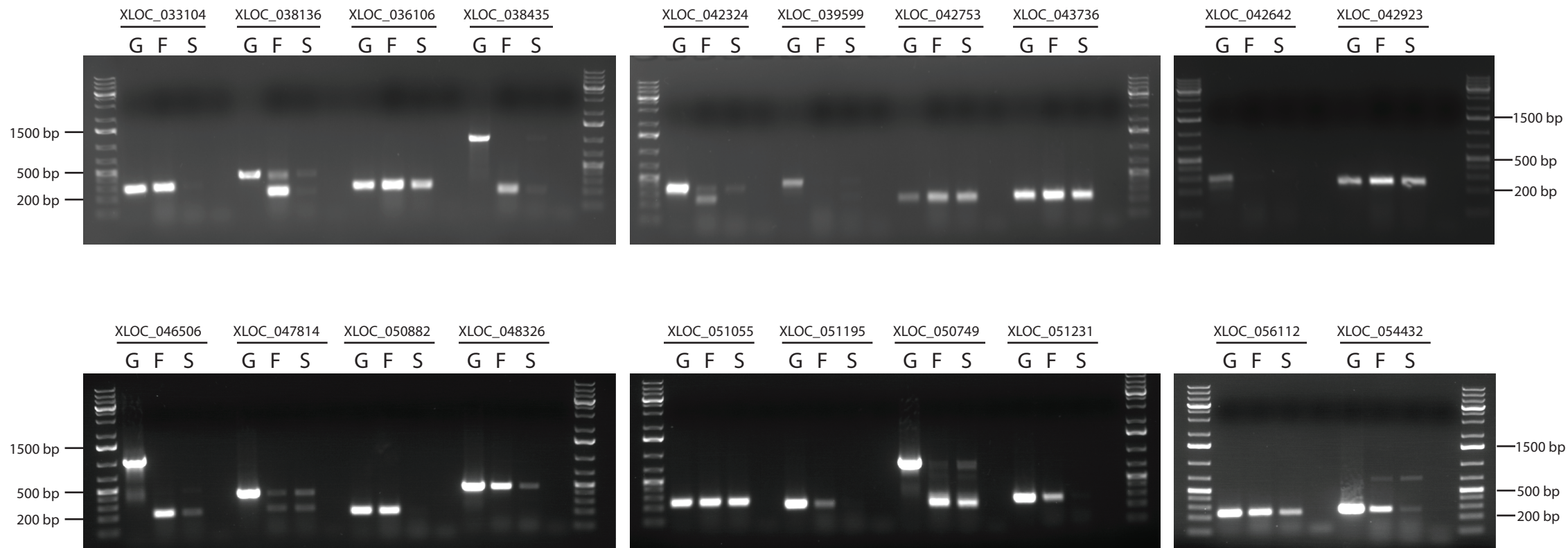

Supplement: Figure S1 — RT-PCR validation of lincRNAs identified in Arabidopsis by Evolinc-I. LincRNA IDs match those found in File S2. G, genomic DNA positive control; F, flower cDNA; S, seedling cDNA. [file Image1.PDF]

A

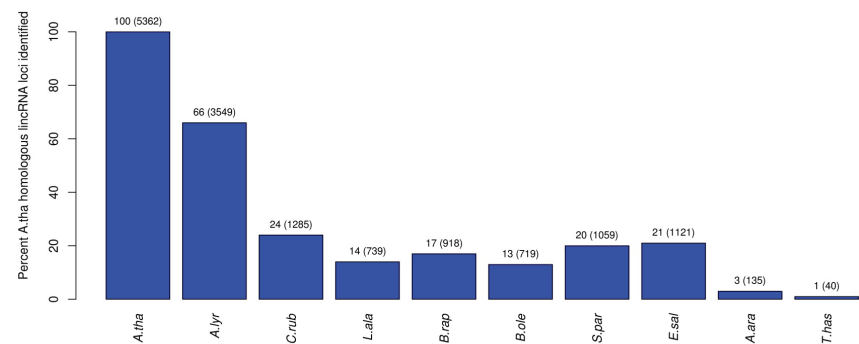

B

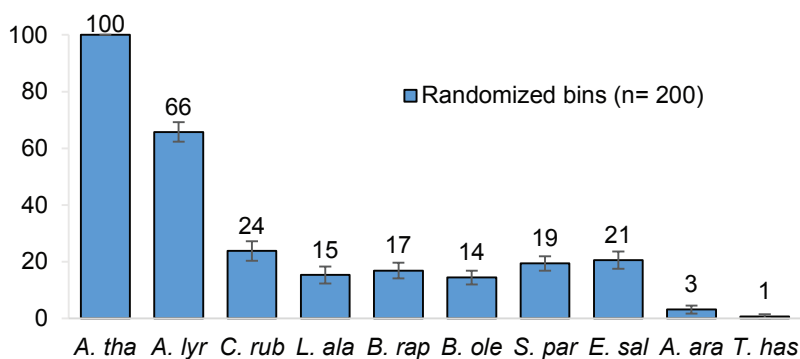

C

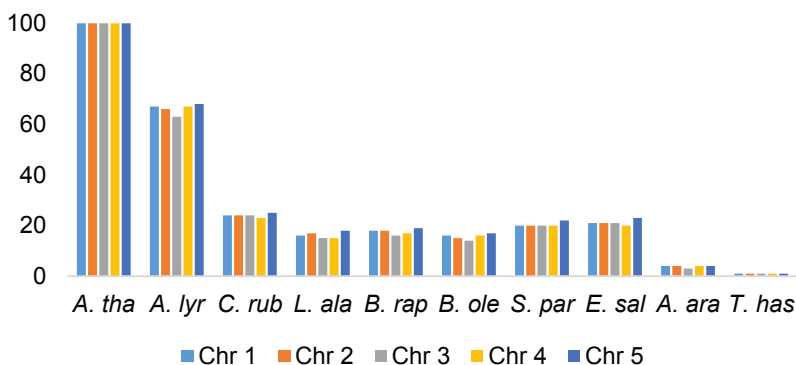

D

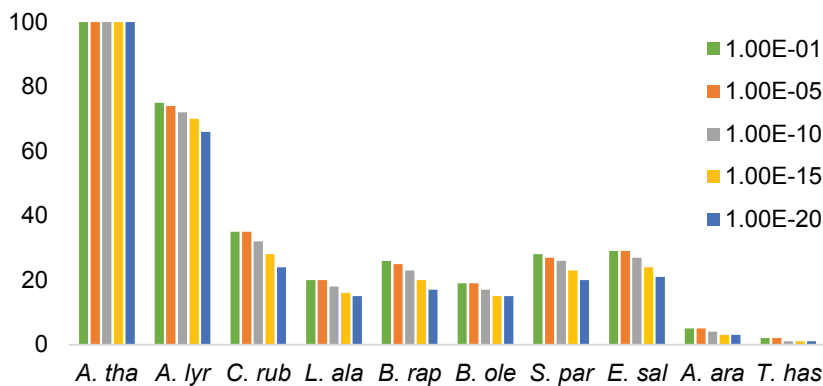

Supplement: Figure S2 — Examining conservation of Liu-lincRNAs in multiple ways with Evolinc-II. (A) Example of the type of bar graph produced by Evolinc-II, in this case for the Liu-lincRNAs at 1E-20. (B) Bar graph of level of lincRNA conservation observed when dividing the Liu-lincRNAs into 27 random bins of 200 lincRNAs each. Standard deviation is based on the difference seen between the 27 bins. (C) Bar graph depicting the level of lincRNA conservation seen when dividing the Liu-lincRNAs by Arabidopsis chromosome (E-cutoff value of 1E-20). (D) Bar graph demonstrating the level of conservation of the Liu-lincRNAs throughout Brassicaceae at different E-cutoff values. [file Image2.PDF]

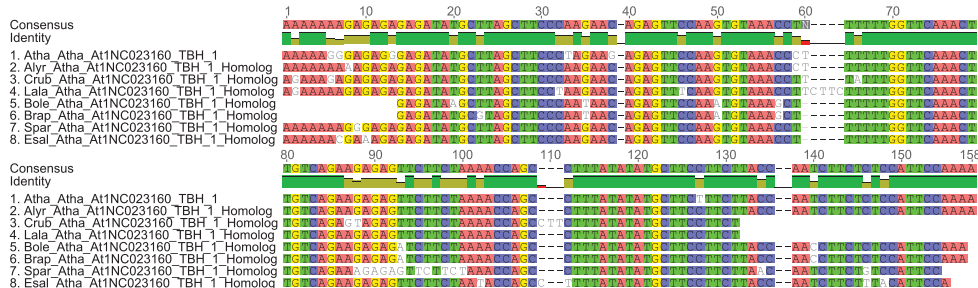

B

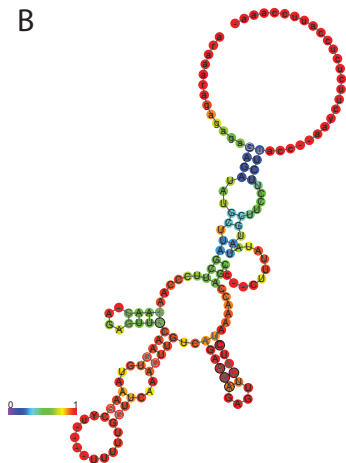

C

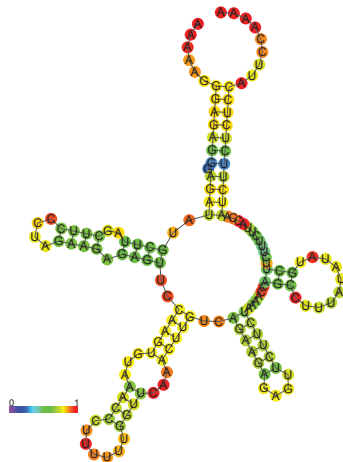

Supplement: Figure S3 — Using At1NC023160 to highlight the structural information that can be gleaned from Evolinc-II. (A) Multiple sequence alignment, generated by MAFFT and visualized within Geneious v7.1 (Kearse et al., 2012). Similar sequences are highlighted, with the consensus sequence shown on top. Nucleotide identity is shown below the consensus sequence, with green representing 100% identity across all sequences. (B) RNAalifold (Lorenz et al., 2011) consensus secondary structure prediction based on multiple sequence alignment in (A). Base-pair probabilities are shown, with red being more probable and blue least probable. (C) RNAfold structure prediction based on the same region as in (B), but limited to just the Arabidopsis sequence. Base-pair probabilities are shown as in (B). [file Image3.PDF]

A

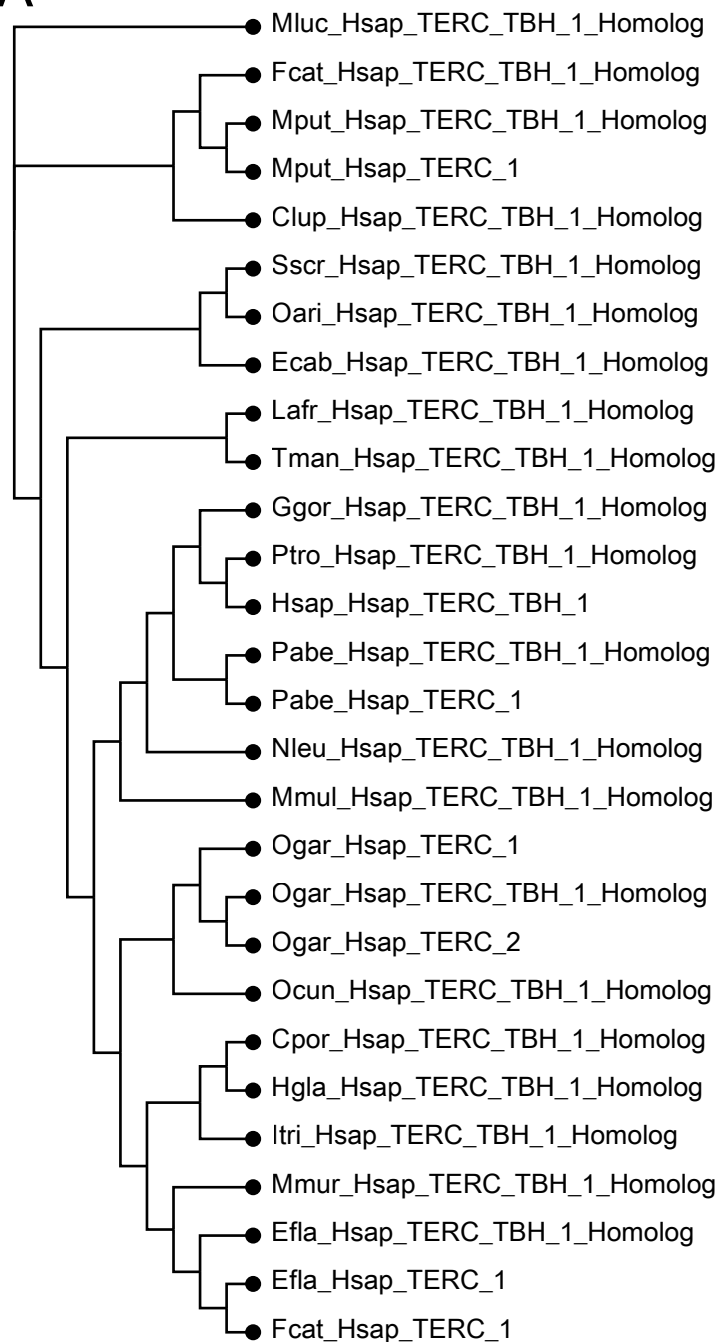

B

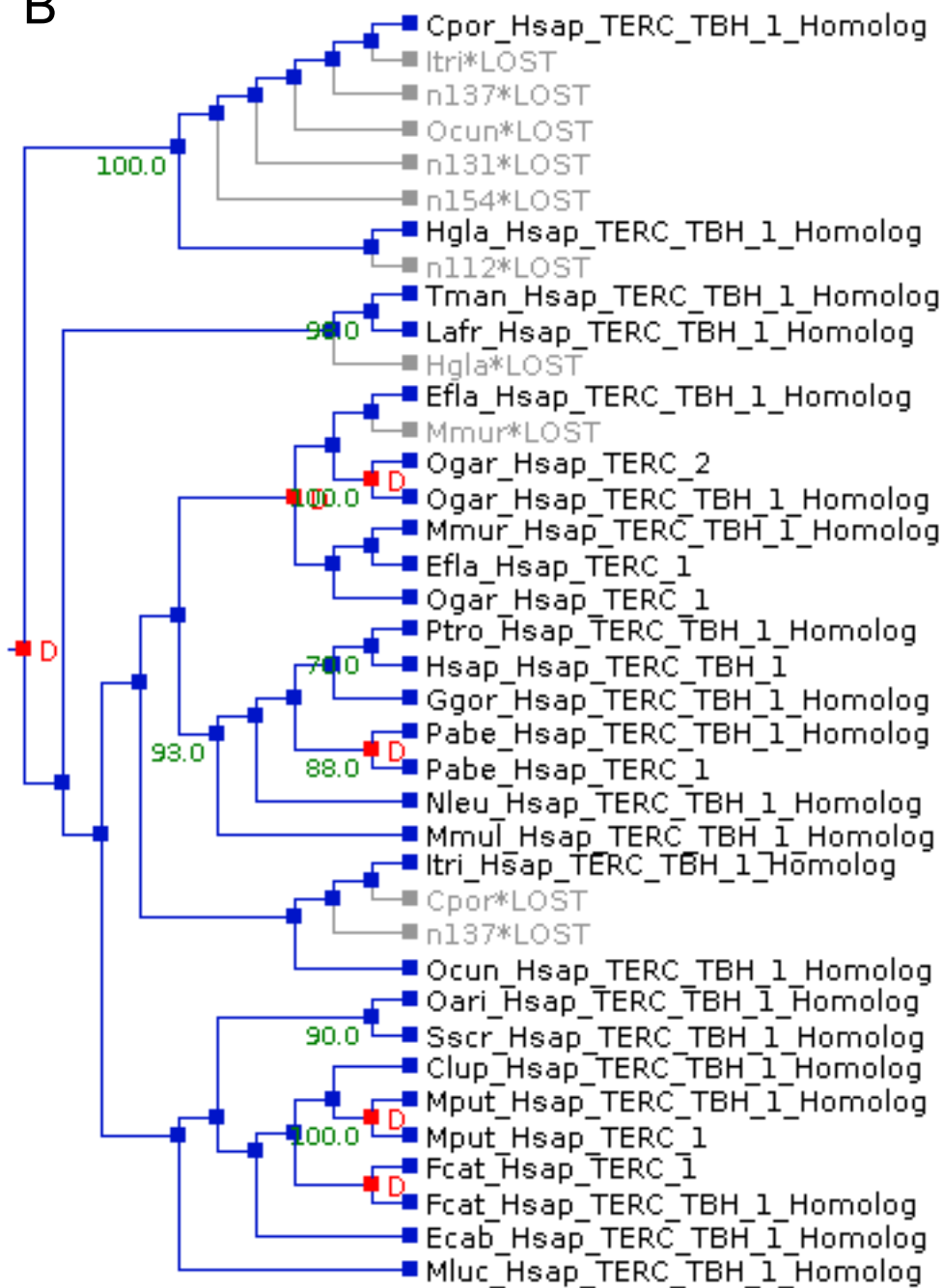

Supplement: Figure S4 — Raw phylogenetic output from Evolinc-II for TERC. (A) A gene tree for the TERC sequence homologs identified in each of the species shown. Sequences without “TBH” indicate paralogs. (B) Notung (Durand et al., 2006) reconciliation of the gene tree shown in (A) to the known species tree. Duplication (red “D”) and loss events (gray “LOST”) are shown. Support for duplication or loss events are indicated by the green numbers at the nodes that represent the predicted origin of those events. [file Image4.PDF]
