# Supplementary material for: Evolinc: A Tool for the Identification and Evolutionary Comparison of Long Intergenic Non-coding RNAs
Source: Front Genet. 2017 May 9;8:52. doi: 10.3389/fgene.2017.00052 (PMC5422434; doi:10.3389/fgene.2017.00052)
Supplement: Table S1 — Percent similarity between transcripts identified following transcript assembly and lincRNA identification. [file Table1.PDF]

	Identified transcription units overlapping between studies		
	Liu et al., 2012	Present study	Percent similarity
ATU	30650	32911	107.3
GATU	370	352	95.1
RCTU	678	643	94.8
lincRNA	278	261	93.8

ATU = Annotated Transcription Unit

GATU = Gene Associated Transcription Unit

RCTU = Repeat Containing Transcription Unit

Nomenclature taken from Liu et al., 2012
